# BrWRKY65, a WRKY Transcription Factor, Is Involved in Regulating Three Leaf Senescence-Associated Genes in Chinese Flowering Cabbage

**DOI:** 10.3390/ijms18061228

**Published:** 2017-06-08

**Authors:** Zhong-Qi Fan, Xiao-Li Tan, Wei Shan, Jian-Fei Kuang, Wang-Jin Lu, Jian-Ye Chen

**Affiliations:** State Key Laboratory for Conservation and Utilization of Subtropical Agro-Bioresources/Guangdong Provincial Key Laboratory of Postharvest Science of Fruits and Vegetables/Key Laboratory of Biology and Genetic Improvement of Horticultural Crops-South China, Ministry of Agriculture, College of Horticulture, South China Agricultural University, Guangzhou 510642, China; ffanzqi@163.com (Z.-Q.F.); tanxl2017@163.com (X.-L.T.); shanwei@scau.edu.cn (W.S.); jfkuang@scau.edu.cn (J.-F.K.); wjlu@scau.edu.cn (W.-J.L.)

**Keywords:** Chinese flowering cabbage, WRKY, postharvest leaf senescence, senescence-associated genes, chlorophyll degradation

## Abstract

Plant-specific WRKY transcription factors (TFs) have been implicated to function as regulators of leaf senescence, but their association with postharvest leaf senescence of economically important leafy vegetables, is poorly understood. In this work, the characterization of a Group IIe WRKY TF, BrWRKY65, from Chinese flowering cabbage (*Brassica rapa* var. parachinensis) is reported. The expression of *BrWRKY65* was up-regulated following leaf chlorophyll degradation and yellowing during postharvest senescence. Subcellular localization and transcriptional activation assays showed that BrWRKY65 was localized in the nucleus and exhibited trans-activation ability. Further electrophoretic mobility shift assay (EMSA) and transient expression analysis clearly revealed that BrWRKY65 directly bound to the W-box motifs in the promoters of three senescence-associated genes (*SAGs*) such as *BrNYC1* and *BrSGR1* associated with chlorophyll degradation, and *BrDIN1*, and subsequently activated their expressions. These findings demonstrate that BrWRKY65 may be positively associated with postharvest leaf senescence, at least partially, by the direct activation of *SAGs*. Taken together, these findings provide new insights into the transcriptional regulatory mechanism of postharvest leaf senescence in Chinese flowering cabbage.

## 1. Introduction

As the final phase of leaf development, senescence is an important biological process accompanying the cessation of photosynthesis, chlorophyll breakdown, leaf proteins degradation, amino acids removal, as well as the mobilization of nitrogen, carbon and other minerals [1,2,3,4]. Although senescence is necessary to maximize a plant’s resource use, premature or abnormal senescence induced by biotic and abiotic stress often causes a decrease in crop yield and produce quality, as well as a decline in shelf-life during postharvest transportation and storage [5,6,7]. Therefore, the underlying regulatory mechanisms of postharvest leaf senescence would be beneficial for many biotechnological applications to prevent postharvest loss [8].

During senescence, leaves undergo a series of programmed molecular changes mediated by senescence-associated genes (*SAGs*) [1,5,9], which in turn, are controlled by transcription factors (TFs) [3,7,9,10,11,12]. The Arabidopsis thaliana genome encodes ~2,000 TFs, among which more than 200 TFs are related to leaf senescence [10,13]. In particular, WRKY, NAC (NAM/ATAF1/2/CUC2), MYB, C2H2 zinc-finger, bZIP, and AP2/ERF represent the largest families of senescence-associated TFs [3,9,10,11,14].

WRKYs form a major family of plant-specific TFs, with more than 70 and 100 members in model species *Arabidopsis thaliana* and rice, respectively [15,16]. WRKYs include one or two highly conserved DNA-binding motifs, termed WRKY domain, characterized by WRKYGQK sequences, following a specific C-terminal zinc-finger motif [15,16]. The first *WRKY* gene is identified from sweet potato [17], and subsequently from many other plants [18]. WRKYs have been suggested to act as major regulators of leaf senescence. For example, the *Arabidopsis thaliana wrky53* mutant displays delayed leaf senescence, while its over-expression results in precocious senescence, demonstrating that WRKY53 is positively involved in leaf senescence [19,20]. Besides WRKY53, other well-characterized WRKYs that are involved in leaf senescence include WRKY6, WRKY22, WRKY33, WRKY53, WRKY54, WRKY70, and WRKY75 from *Arabidopsis thaliana* [3,9,11,12,21], OsWRKY23, OsWRKY42, OsWRKY80, and OsWRKY26 from rice [22,23,24], and TaWRKY7 from wheat [25]. To date, however, little is known about the molecular aspects of senescence-associated WRKY TFs in non-model plants, especially in economically important leafy vegetables.

The *Brassica rapa* L. comprises many economically important worldwide cultivated leafy vegetables, such as Chinese cabbage (*B. rapa* ssp. *pekinensis*), bok choy (*B. rapa* ssp. *chinensis*), and Chinese flowering cabbage/choy sum (*B. rapa* ssp *parachinensis*) [26]. These leafy vegetables are known for their nutraceutical and health-promoting properties [27]. However, harvested leafy vegetables wilt, senesce and perish rapidly, leading to short shelf-life and substantial losses. Previous studies have examined several physiological parameters and identified some *SAGs* genes related to postharvest leaf senescence of leafy vegetables, and established storage technologies that delay leaf senescence [28,29,30,31]. Nevertheless, transcriptional regulatory mechanism(s) underpinning WRKY TF-regulated postharvest leaf senescence in leafy vegetables remains unexplored. Here, a leaf senescence-inducible WRKY TF BrWRKY65, is isolated and characterized in Chinese flowering cabbage. We provide evidences that BrWRKY65 may be positively associated with postharvest leaf senescence in Chinese flowering cabbage by the activation of three *SAGs* genes including *BrNYC1* and *BrSGR2* related to chlorophyll degradation, and *BrDIN1*.

## 2. Results and Discussion

### 2.1. Physiological Changes During Chinese Flowering Cabbage Postharvest Leaf Senescence

**Figure 1:**
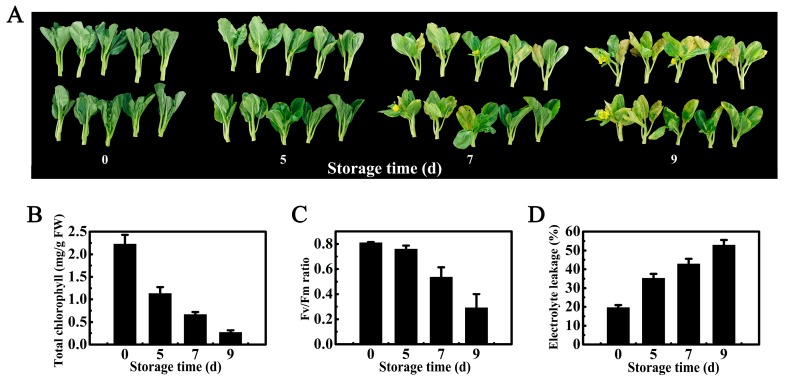
(**A**) Appearance of Chinese flowering cabbage during 9 days of storage at 15 °C; (**B**–**D**) Changes in total chlorophyll content, Fv/Fm ratio, and relative electrolyte leakage during postharvest leaf senescence. Data represent mean values from three biological replicates (±S.E.).

Leaf yellowing due to a rapid breakdown of chlorophyll is an obvious symptom of senescence in cabbages [29,30]. As shown in Figure 1A, the Chinese flowering cabbage leaves started yellowing after 5 days of storage at 15 °C, and it got severe by day 9. Chlorophyll content and relative electrolyte leakage are used as markers for assessing the severity of senescence progression (Lin et al. 2015)[32]. Correlative to the leaf yellowing, total chlorophyll content (Figure 1B) and Fv/Fm ratio (Figure 1C) declined continuously such that their values on day 9 were just 11.3% and 37.4% of day 0, respectively (Figure 1B,C). In contrast, relative electrolyte leakage of cabbage leaves increased gradually during postharvest senescence (Figure 1D).

### 2.2. Isolation and Bioinformatics Analysis of BrWRKY65

**Figure 2:**
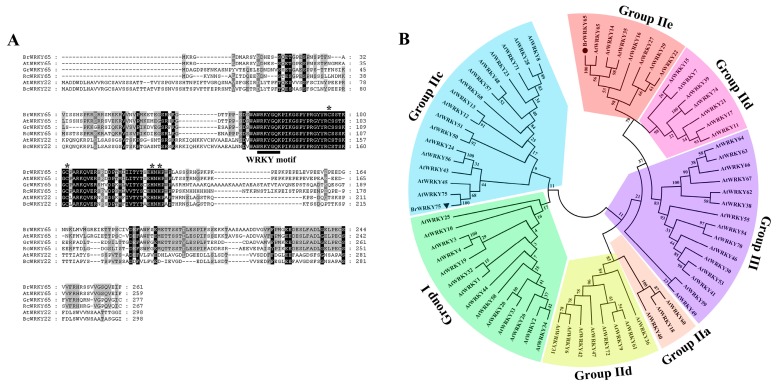
Bioinformatics analysis of BrWRKY65. (**A**) Multiple alignment of BrWRKY65 with *Arabidopsis thaliana* AtWRKY22 (XP_002872898.1) and AtWRKY65 (NP_174222.2), Pak-choi BcWRKY22 (NP_001288962.1), cotton GrWRKY65 (NP_001313892.1), and castor bean RcWRKY65 (XP_002522247.1). Black and gray shading indicate identical and similar amino acids respectively. The WRKY motif is underlined and the zinc-finger structures are indicated by asterisks; (**B**) A phylogenetic tree of *Arabidopsis thaliana* WRKYs and BrWRKY65. Three major groups and seven sub-families of WRKYs are presented in different colors. BrWRKY65, along with AtWRKY65, AtWRKY22, and AtWRKY35 were classified into Group IIe. MEGA5.0 was applied to construct the phylogenetic tree with default parameters.

Plant-specific WRKY TFs play a central role in regulating leaf senescence [3,9,12,33]. Although 145 WRKY TFs are found in *Brassica rapa* plants such as Chinese cabbage [34,35], and a senescence-related Group IIc WRKY TF BrWRKY75 is preliminarily characterized in our previous study [36], the mechanism(s) of senescence-associated WRKY TFs involved in regulating leaf senescence in Chinese flowering cabbage remains unclear. However, in the present work, searching our RNA-seq transcriptome database, a full-length *WRKY* gene (NP_001306245.1) showed increased expression during postharvest cabbage leaf senescence. The full-length of this *WRKY* was cloned and homology search indicated that it exhibited the highest identity with AtWRKY65 (85%); so it was designated as *BrWRKY65*. The ORF of *BrWRKY65* is 786 bp in length and encodes a polypeptide of 262 amino acids. The calculated molecular weight and *p*I of BrWRKY65 is 29 kDa and 5.41 respectively. Multiple alignments of BrWRKY65 revealed that it possesses a highly conserved WRKY domain, with WRKYGQK sequence, which is the defining character of WRKY proteins [15], as well as one putative zinc-finger motif (C-X5-CX23-H-X1-H) at the C-terminus (Figure 2A).

According to the number of WRKY domains and structure of the zinc-finger motif, WRKY TFs are clustered into three major groups (I-III). Group II can be further divided into five subfamilies (IIa-e) [15,16]. A phylogenetic tree that was constructed using BrWRKY65, BrWRKY75, and Arabidopsis WRKYs showed that BrWRKY75 belonged to Group IIc, while BrWRKY65 belonged to Group IIe, along with AtWRKY65, AtWRKY22, and AtWRKY35 (Figure 2B), of which AtWRKY22 positively regulates dark-induced leaf senescence [21], suggesting the possible involvement of BrWRKY65 in cabbage leaf senescence.

### 2.3. BrWRKY65 Is Localized to the Nucleus and Acts as a Transcription Activator

**Figure 3:**
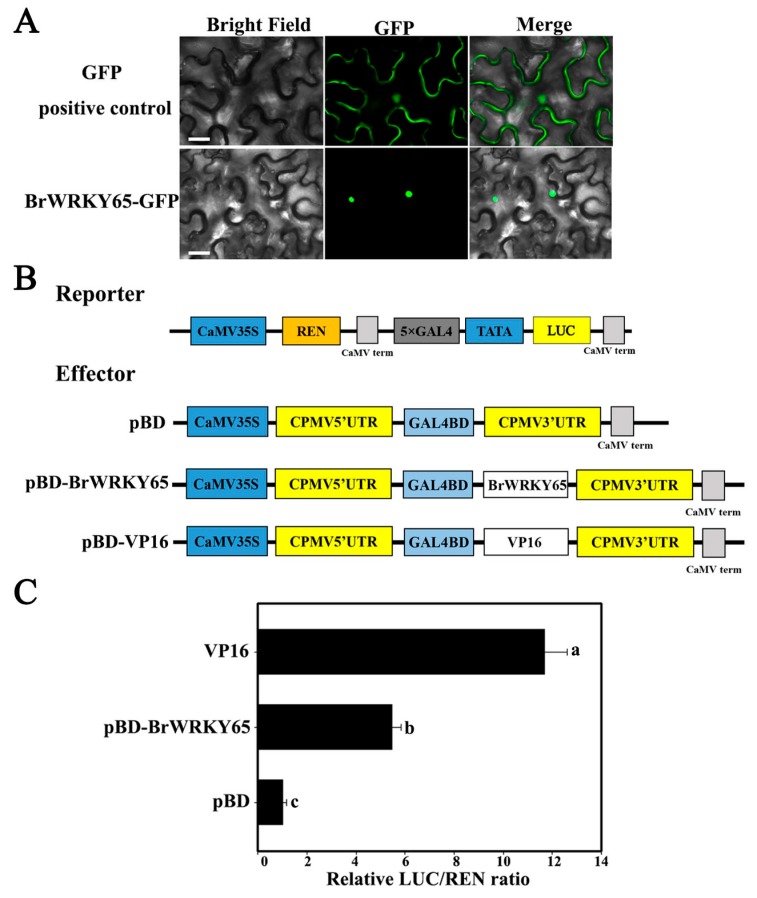
(**A**) Nuclear localization of BrWRKY65 in tobacco leaves. The fusion protein (BrWRKY65-GFP) and positive control were transiently expressed in tobacco leaves respectively via *Agrobacterium tumefaciens* strain GV3101. GFP signals were captured with a fluorescence microscope after 48 h of injection. Images were photographed in a dark-field for GFP, while the outline of the cell and the merged were taken in a bright field. Bars, 25 μm; (**B**) Schematics of the reporter and effector constructs. The firefly luciferase (LUC) was drove by the minimal TATA box of the CaMV 35S promoter plus five copies of the GAL4 binding element (5× GAL4), and the CaMV 35S-driving Renilla luciferase (REN) at the same vector was used as an internal control. *BrWRKY65* fused with the yeast GAL4 DNA-binding domain (GAL4BD) driven by CaMV 35S, were adopted as the effector; (**C**) Transcriptional activation activity of BrWRKY65. The trans-activation ability of BrWRKY65 is revealed by the LUC/REN ratio. The ratio of LUC/REN of the empty pBD vector is used as the calibrator (set as 1). At least six independent repeats were assayed for each pair. Compared with the pBD, significant differences at the level of *p* < 0.05 analyzed by the Student’s *t*-test, are indicated by different letters above the bar.

As TFs, WRKYs are usually nuclear proteins and possess transcriptional activity [33,37,38,39]. Previously, we also found that Chinese flowering cabbage BrWRKY75 was a nuclear protein with transcriptional repression activity [36]. To investigate the subcellular location of BrWRKY65, it was fused with GFP and got transiently expressed in tobacco leaves. As shown in Figure 3A, GFP signal of BrWRKY65 fusion protein was detected in the nucleus of tobacco cells, while the positive control GFP fluorescence was observed around the cytoplasm and the nucleus. The transcriptional activity of BrWRKY65 was analyzed through the dual-luciferase reporter system, also in tobacco leaves. For this assay, LUC reporter is fused with 5× GAL4 DNA-binding elements plus TATA box and REN driven by the 35S promoter is used as the internal control (Figure 3B). Compared with the negative control pBD, both the transcriptional activator control VP16 and BrWRKY65 significantly increased the values of LUC/REN ratio (Figure 3C). These data suggest that BrWRKY65 may act as a transcriptional activator in the nucleus.

### 2.4. Expression Patterns of BrWRKY65, BrNYC1, BrSGR1, and BrDIN1 during Chinese Flowering Cabbage Postharvest Leaf Senescence

**Figure 4:**
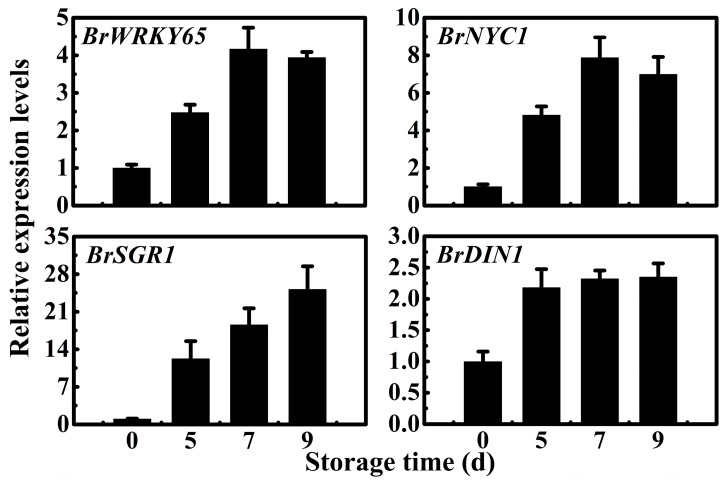
Expression patterns of *BrWRKY65*, *BrNYC1*, *BrSGR1*, and *BrDIN1* during Chinese flowering cabbage postharvest leaf senescence. Transcript levels of each gene at 0 d of storage were set as 1. Data represents the mean ± S.E. of three biological replicates.

To further confirm the possible association of BrWRKY65 with cabbage postharvest leaf senescence, its expression was examined by qRT-PCR. Consistent with its trend in our RNA-seq database, *BrWRKY65* transcript level increased during senescence, reaching about 4.2- and 3.8-fold of the initial level at day seven and nine, respectively (Figure 4). In addition, as leaf yellowing is due to chlorophyll degradation [29,32,40,41,42,43], and dark-inducible (*DIN*) genes are induced in dark-adapted and senescing leaves [44,45], which have been used previously as markers to characterize senescence-associated responses. Therefore, we further searched the *SAG* genes in our RNA-seq transcriptome database, and three *SAG*s such as *BrNYC1* (NON-YELLOW COLORING1/Chl *b* reductase) (XP_009139641.1) and *BrSGR1* (STAY-GREEN1) (XP_018512704.1) associated with chlorophyll degradation, and *BrDIN1* (XP_009150416.1) were selected. Their expressions were also analyzed. As can be seen in Figure 4, as expected, similar to *BrWRKY65*, the expression of *BrNYC1*, *BrSGR1*, and *BrDIN1* were all up-regulated during cabbage postharvest leaf senescence.

### 2.5. BrWRKY65 Directly Binds to the W-Box Elements on the Promoters of BrNYC1, BrSGR1, and BrDIN1

**Figure 5:**
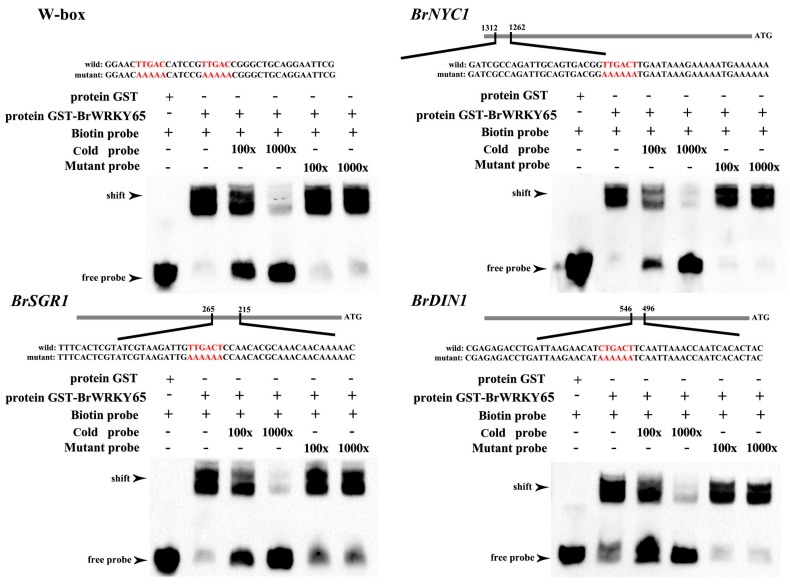
Electrophoretic mobility shift assay (EMSA) of BrWRKY65 binding to the W-box element and promoters of *BrNYC1*, *BrSGR1*, and *BrDIN1* containing W-box element. The probe sequences corresponding to each of the target gene promoters are shown, with red letters representing the W-box and the mutant W-box. The purified recombinant GST-BrWRKY65 protein was incubated with probes, and the protein–DNA complexes were separated on native polyacrylamide gels. GST protein alone was used as the negative control. − represents absence, while + represents presence. Unlabeled or mutant probes at different concentrations (from 100 to 1000 times) were added to the reaction mixture for competition and testing binding specificity. Arrows indicate the position of shifted bands.

Numerous studies reveal that WRKY TFs control their target gene expressions by binding to the typical *cis*-element W-box with a core sequence (C/T)TGAC(C/T) in the promoter [16,33,39,46]. We scanned the promoter regions of *BrNYC1*, *BrSGR1*, and *BrDIN1*, and found that they all contain putative W-box motifs (Text S1). In addition, qRT-PCR results revealed that *BrWRKY65*, *BrNYC1*, *BrSGR1*, and *BrDIN1* showed similar expression trends during leaf senescence (Figure 4). Thus, it could be speculated that *BrNYC1*, *BrSGR1*, and *BrDIN1* might be targets of BrWRKY65. The direct binding of BrWRKY65 protein to *BrNYC1*, *BrSGR1*, and *BrDIN1* promoters was verified by an Electrophoretic mobility shift assay (EMSA). Purified recombinant glutathione S-transferase (GST)-BrWRKY65 fusion protein was successfully obtained via prokaryotic expression (Figure S1). As expected, the GST-BrWRKY65 fusion protein was able to bind the biotin-labeled probes containing the W-box motif derived from *BrNYC1*, *BrSGR1*, and *BrDIN1* promoters and caused mobility shifts. However, the mobility shift was effectively abolished when unlabeled *BrNYC1*, *BrSGR1*, or *BrDIN1* promoter fragment used as a cold competitor was added, in a dose-dependent manner, but not by the mutated W-box probes (Figure 5). The mobility shift was also not observed when the biotin-labeled probes were incubated with GST alone (Figure 5), indicating that the binding of BrWRKY65 to the *BrNYC1*, *BrSGR1*, or *BrDIN1* promoter is specific. While further experiments such as chromatin immunoprecipitation (ChIP) assays will be needed to confirm the bindings of BrWRKY65 to *BrNYC1*, *BrSGR1*, and *BrDIN1* in vivo.

### 2.6. BrWRKY65 Activates the Expressions of BrNYC1, BrSGR1, and BrDIN1

**Figure 6:**
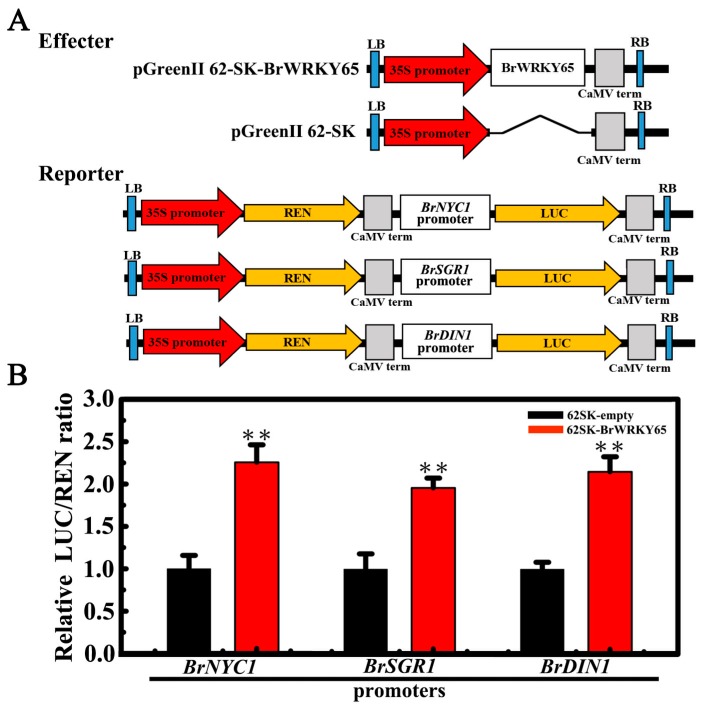
Transient dual-luciferase reporter assay showing the trans-activation of *BrNYC1*, *BrSGR1*, and *BrDIN1* by BrWRKY65. (**A**) Diagrams of the reporter and effector constructs; (**B**) BrWRKY65 trans-activated *BrNYC1*, *BrSGR1*, and *BrDIN1* promoters. The ratio of LUC/REN of the empty vector (62-SK) plus promoter was considered as a calibrator (set as 1). The activation is indicated by the ratio of LUC to REN. Data represents the mean ± S.E. of six independent repeats. ** indicates statistically significant differences at the level of *p* < 0.01 tested by Student’s *t*-test.

Since BrWRKY65 is a transcription activator (Figure 3C), the trans-activation of *BrNYC1*, *BrSGR1*, and *BrDIN1* by BrWRKY65 was further determined through transient dual-luciferase assays in tobacco leaves. The *BrNYC1*, *BrSGR1*, and *BrDIN1* promoters with 1331, 533, and 1229 bp, respectively, were fused with the LUC reporter. CaMV 35S-driving REN reporter in the same vector was used as an internal control to normalize the expression of each reporter (Figure 6A). BrWRKY65 inserted into the pGreenII 62-SK was adopted as an effector, and the empty pGreenII 62-SK vector was included as a control (Figure 6A). The respective reporter and effector plasmids were co-expressed in tobacco leaves and the LUC/REN ratio was detected. As shown in Figure 6B, compared with the empty control (pGreenII 62-SK), overexpression of BrWRKY65 with *BrNYC1*, *BrSGR1*, or *BrDIN1* promoter significantly increased the value of the LUC/REN ratio. The results clearly suggest that BrWRKY65 activated the transcription of *BrNYC1*, *BrSGR1*, and *BrDIN1*, revealing that BrWRKY65 may function as a positive regulator of postharvest leaf senescence of Chinese flowering cabbage. While it should be pointed out that targeted transgenic research are required to fully unravel the biological function of BrWRKY65 in regulating postharvest leaf senescence of Chinese flowering cabbage. The previously identified BrWRKY75 exhibited transcriptional repression activity and was also up-regulated during leaf senescence [36]. Accordingly, whether BrWRKY65 and BrWRKY75 act together or individually to regulate *SAGs* is an interesting research issue in the future. Moreover, it has been reported other types of Arabidopsis TFs, such as ABSCISIC ACID INSENSITIVE3 (ABI3) [47], ABI5, ENHANCED EM LEVEL (EEL) [48], phytochrome-interacting factor 4 (PIF4) [49], MYC2/3/4 [50], ANAC072 [43], and ERF [51], are reported to activate genes related to chlorophyll degradation such as *NYE1* (also known as *SGR1*), *NYC1*, and *PAO* (pheophorbide a oxygenase) via directly binding to their promoters, leading to accelerated chlorophyll degradation during leaf senescence, seed or fruit degreening. Intriguingly, Arabidopsis ETHYLENE INSENSITIVE3 (EIN3) and ORE1/NAC2 establish a network controlling ethylene-induced leaf senescence via directly targeting *NYE1*, *NYC1*, and *PAO* [52]. More recently, abscisic acid (ABA)-responsive element (ABRE)-binding TFs ABF2, ABF3, and ABF4 from *Arabidopsis* are found to speed ABA-mediated chlorophyll degradation and leaf senescence by the trans-activation of *NYE1*, *NYE2*, *NYC1*, *PAO*, *SAG12*, and *SAG29* [42]. Collectively, these results imply the existence of a complex regulatory network of chlorophyll degradation and leaf senescence involving multiple TFs. Therefore, whether BrWRKY65 coordinates with these TFs in regulating postharvest leaf senescence in Chinese flowering cabbage needs to be investigated in the future.

## 3. Materials and Methods

### 3.1. Plant Materials and Samples

Chinese flowering cabbages (*Brassica rapa* var. parachinensis) were obtained from a local commercial vegetable farm near Guangzhou, southern China. After pre-cooling, the harvested cabbages were transported to the lab immediately. Only cabbages with no mechanical damage and uniform appearance were chosen. The selected cabbages were placed into plastic baskets (20 per box) packing with 0.04 mm thickness polyethylene perforated plastic bags. The packed cabbages were then stored in the incubators at 15 °C. Samples were taken at 0, 5, 7, and 9 days of storage, and the second leaf from the bottom of the cabbages was collected. The collected leaves were sliced into small pieces, frozen in liquid nitrogen and stored at −80 °C until analysis.

### 3.2. Leaf Senescence Evaluations

Leaf senescence was assessed using the following three parameters: total chlorophyll content, chlorophyll fluorescence and relative electrolyte leakage. The total chlorophyll content per fresh weight of leaf tissue was estimated spectrophotometrically after extraction in 80% acetone as previously described [23,29]. Chlorophyll fluorescence was measured using the ‘Fv/Fm’ mode on an OS-500 modulated Fluorometer (OPTI Sciences, Boston, MA, USA). The Fv/Fm is a ratio of the variable fluorescence divided by the maximum fluorescence value. The Fv/Fm ratio was measured after cabbage leaves were placed in the dark for 30 min at room temperature [53]. Relative electrolyte leakage was defined as L_t_/L_0_ and expressed as a percentage, which was measured using a conductivity meter [54].

### 3.3. RNA Extraction, Gene Isolation, and Sequence Analysis

Total RNA was extracted from Chinese flowering cabbage leaves using the RNeasy Mini kit (Qiagen, Hilden, Germany) following the manufacturer’s protocol. The quality and integrity of total RNA was monitored by running ~1 μg in a formamide denaturing gel, and using a spectrophotometer to confirm total RNA quantity. A PrimeScript^TM^ RT reagent Kit with gDNA Eraser (Takara, shiga, Japan) was applied to synthesize cDNAs, following the manufacturer’s instructions. 

Based on our RNA-seq database and genome of Chinese cabbage *chiifu* in BRAD (http://brassicadb.org/brad/), a WRKY showing high sequence homology to *Arabidopsis thaliana* WRKY65, named *BrWRKY65* (NP_001306245.1), was found to be up-regulated during postharvest leaf senescence. BrWRKY65 was cloned, sequenced (primers are listed in Table S1), and subjected to a homology search in the NCBI database. The theoretical isoelectric point (*p*I) and mass value for BrWRKY65 protein were predicted at the online website (http://web.expasy.org/compute_pi/). Multiple alignments were performed using CLUSTALW (version 1.83) and GeneDoc softwares. The phylogenetic tree of WRKY proteins was constructed using the MEGA5.0 software with the Neighbor–Joining method.

### 3.4. Gene Expression Analysis by qRT-PCR

Quantitative RT-PCR (qRT-PCR) was conducted as previously described [55]. PCR reactions were carried out with the GoTaq^®^ qPCR Master Mix Kit (Promega, Madison, WI, USA) on a Bio-Rad CFX96 Real-Time PCR System. Data were normalized to reference gene *EF-1-α* (GO479260) [56]. All qRT-PCRs were normalized using the cycle threshold (*C*_t_) value corresponding to the reference gene. Primers used for qRT-PCR are listed in Table S1.

### 3.5. Analysis of BrWRKY65 Subcellular Localization

The complete Open Reading Frame (ORF) of BrWRKY65 was amplified and inserted into the pEAQ-GFP vector (primers are listed in Table S1). The pEAQ-BrWRKY65-GFP plasmids were introduced into the *Agrobacterium tumefaciens* strain GV3101 using Gene PulserXcell^TM^ Electroporation Systems (Bio-Rad, Hercules, CA, USA). The *Agrobacterium* harboring pEAQ-BrWRKY65-GFP was injected into the abaxial side of 4- to 6-week-old tobacco (*Nicotiana benthamiana*) leaves using a 1-mL syringe without a needle as described previously [57,58]. pEAQ-GFP was employed as the positive control. The GFP signal was visualized with an Axioskop 2 Plus fluorescence microscope (Zeiss, Jena, Germany) after 48 h of infiltration.

### 3.6. Promoter Isolation and Analysis

Genomic DNA was prepared from Chinese flowering cabbage leaves using the CTAB-based methods [59]. The promoters of three *SAGs* including *BrNYC1*, *BrSGR2*, and *BrDIN1* were obtained using a Genome Walker Kit (Clontech, Mountain View, CA, USA) with nested PCR (specific primers are listed in Table S1). The Plant-CARE database (http://bioinformatics.psb.ugent.be/webtools/plantcare/html/) was employed to predict the conserved *cis*-element motifs presented in promoters. 

### 3.7. Recombinant Protein Expression, Purification and Electrophoretic Mobility Shift Assay (EMSA)

The GST-BrWRKY65 expression vector was constructed with pGEX-4T-1 (GE Healthcare Life Sciences (China), Beijing, China) and then transformed into *Escherichia coli* strain BM Rosetta (DE3). GST-BrWRKY65 protein expression was induced by 1mM isopropyl thio-β-d-galactoside (IPTG) at 30 °C for 6 h, and the recombinant fusion protein was purified using Glutathione-Superflow Resin (Clontech, Mountain View, CA, USA) according to the manufacturer’s protocol.

The fragments of ~60 bp containing putative WRKY binding region in the promoters of *BrNYC1*, *BrSGR2*, and *BrDIN1* were labeled with biotin at the 5’ end. An EMSA was performed essentially using the LightShift Chemiluminescent EMSA Kit (Thermo Scientific, Rockford, IL, USA) as our previous studies described [58,60]. Purified GST-BrWRKY65 fusion protein was incubated with biotin-labeled DNA fragments, and the protein-DNA complexes were separated by SDS-PAGE following detection on a ChemiDoc™ MP Imaging System (Bio-Rad, Hercules, CA, USA) by the chemiluminescence method. A 100- and 1000-fold molar excess of unlabeled DNA fragments with the same or mutant sequences were used as competitors, and the GST protein alone was used as the negative control. The primers used in the EMSA assay are listed in Table S1.

### 3.8. Dual-Luciferase Transient Expression Analysis in Tobacco Leaves

The transcriptional activity of BrWRKY65 was assayed using the dual-luciferase transient expression system in tobacco leaves. The reporter vector was modified from the pGreenII 0800-LUC vector [61]. The firefly luciferase (LUC) was driven by the minimal TATA box of the CaMV 35S promoter plus five copies of the GAL4 binding element (5× GAL4), and the Renilla luciferase (REN) driven by CaMV 35S at the same vector was used as an internal control. The full-length coding sequence of BrWRKY65 was fused with the yeast GAL4 DNA-binding domain (GAL4BD) as the effector, driven by CaMV 35S. To assess the trans-activation of BrWRKY65 to the *BrNYC1*, *BrSGR2*, and *BrDIN1* promoters, these promoters were inserted into pGreenII0800-LUC vector, while *BrWRKY65* was cloned into the pGreenII 62-SK vector as the effector [61]. Primers for all constructs are listed in Table S1.

The constructed reporter and effector plasmids were transiently co-expressed in tobacco leaves as described above. After 48 h of infiltration, dual-luciferase assay kit (Promega (Beijing) Biotech Co., Ltd., Beijing, China) was adopted to detect LUC and REN luciferase activity on a Luminoskan Ascent Microplate Luminometer (Thermo Scientific, Rockford, IL, USA) according to the manufacturer’s instructions. The transcriptional activity of BrWRKY65, and trans-activation of BrWRKY65 to each promoter was indicated by the LUC to REN ratio. At least six independent repeats were measured for each pair.

### 3.9. Statistics

Experiments were performed according to a complete randomized design. Data in figures are expressed as the mean ± standard errors (S.E.) of at least three independent replicates. The data were statistically analyzed by applying Student’s *t*-test.

## 4. Conclusions

In conclusion, a postharvest leaf senescence-inducible transcriptional activator, BrWRKY65, from Chinese flowering cabbage is isolated and characterized. Furthermore, BrWRKY65 binds to the promoters of three *SAG* genes, and activates their expressions. Overall, our work provides novel information about the transcriptional regulation of leaf senescence in commercially important leafy vegetables, such as Chinese flowering cabbage.

## Supplementary Materials

Supplementary materials can be found at www.mdpi.com/1422-0067/18/6/1228/s1.
