# Supplementary material for: BrWRKY65, a WRKY Transcription Factor, Is Involved in Regulating Three Leaf Senescence-Associated Genes in Chinese Flowering Cabbage
Source: Int J Mol Sci. 2017 Jun 8;18(6):1228. doi: 10.3390/ijms18061228 (PMC5486051; doi:10.3390/ijms18061228)
Supplement: Supplementary file 1 [file ijms-18-01228-s001.pdf]

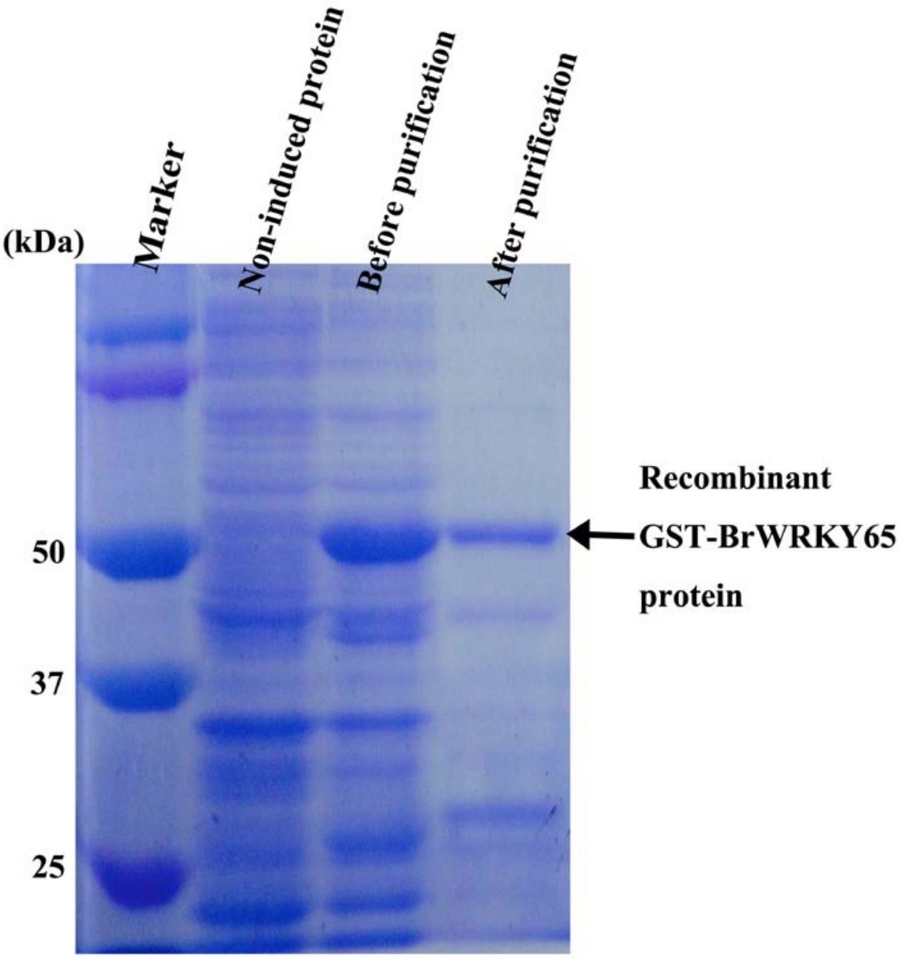

Figure S1. SDS-PAGE gel stained with coomassie blue demonstrating affinity purification of the recombinant GST-BrWRKY65 protein used for the EMSA assay.

Table S1. Summary of primers used in this study.

Assay	Primer sequence			Restriction Site
		Forward primer(5'-3')	Reverse primer(5'-3')	
RT-qPCR	*BrWRKY65*	CGGCACAAAGGAGACACA	TGCTTCTCTCGACTTGCTTC	
	BrNYC1	GATGTCTGGAACACCGCCTC	TTCTCACGCTTTCTCCGTCTTT	
	BrSGR1	CAAAGCTCCCAAGAACTTACACTC	AGATTCTTGTAGCTCAGGGTAACT	
	BrDIN1	GAAACTACCAACGGCGACAAGAAG	CCGGTGTCCTCACGTCCAGA	
Subcellular localization	*BrWRKY65-GFPFor*	ttctgcccaaatcgcg ATGAAGCGAGGTCTAGATAT		*NruI*
	BrWRKY65-GFPRev	tagtcataccggtcgc AAAGATCTCAACCTGTGACCCAAC		*NruI*
Fusing GST	*pGEX-4T-1-BrWRKY65-For*	ggttccgcgtggatcc ATGAAGCGAGGTCTAGATAT		*BamHI*
	pGEX-4T-1-BrWRKY65-Rev	agtcacgatgcggccgc TCAAAAGATCTCAACCTGTGA		*NotI*
Dual LUC assay	*pBD-BrWRKY65For*	tcgccgaccggtaggcct ATGAAGCGAGGTCTAGATAT		*Stu I*
	pBD-BrWRKY65Rev	aaccagagttaaaggcct AAAGATCTCAACCTGTGACCCAAC		*Stu I*
	62sk-BrWRKY65For	tagaactagtggatcc ATGAAGCGAGGTCTAGATAT		*BamHI*
	62sk-BrWRKY65Rev	cggtatcgataagctt TCAAAAGATCTCAACCTGTGACCC		*HindIII*
	0800-BrNYC1pro1For	tatagggcgaattgg GATTTAGAACAAGCCAGATCGCCA		*KpnI*
	0800-BrNYC1proRev	ttggcgtcttccatgg AGCTAAACCAGAGAGTGAGAAGAGGAG		*NcoI*
	0800-BrSGR1proFor	tatagggcgaattgg ATAGATAAGTTCCGACCGAAGCC		*KpnI*
	0800-BrSGR1proRev	ttggcgtcttccatgg CTCTGCTTCGTTTCAATATCAGTA		*NcoI*
	0800-BrDIN1proFor	tatagggcgaattgg GCAGACACTAGGCAATAAAGGCAC		*KpnI*
	0800-BrDIN1proRev	ttggcgtcttccatgg TATACAGACACAAAGCCAAAGGATA		*NcoI*

Text S1. Promoter nucleotide sequences of *BrNYC1*, *BrSGR1 and BrDIN1*. W-box (C/T)TGAC(C/T) is marked by red and boxed. Translation start site (ATG) was shown in yellow box.

BrNYC1 (XP_009139641.1) promoter
GATTTAGAACAAGCCAGATCGCCAGATTGCAGTGACGGTTGACTTGAATAAAGAAAAATGAAAAAAGCAGATAAA
TGCAAAGTTTCTAAGATGAGGATAAGAAAAACTCTCCGCAAAATTAAGAAAGACGGCCGACCTTGGAGGTGTATA
CAAAGACACCTTAGGCCAAGAGGACGAGGGACATAATTTTTTTAGAAACTTAGCACTAGAGTAATTTTAGGCAAT
TTTGTTGTTGTTATCGAACTGCGACTCAAAGAGATTTAAGGCTTCAGATGGTTAGAACTATAGAACTCACTCACAG
CTCTTGCGACCGTAGCTTTTACAACTTGTTGTAACAATCACAACGCTCTTAAGCGGATTTGAAATAGGAGCTGTTCT
TCATTGATTTTTCTTTTCTGTTCGGACTGGATAAAAATAAATTTTCCTTCGTCTCTCCTCTTTTACTTTGATGTGTGTT
TGATACTTTGATTCAAGAGACACCACCACGTCGTCGCTGTCTCGTTGATCTTCTTCTGTTTTGTTGGTCATGCTCAC
TCTCTATTTCCAGAGAAATCAATCTTTGCAATTAAGAAGATGTTCCGGCAAAGTATTTTGAAAGGATAGAGTGTTGA
GATTTGCATAATAGTTTCTCTGATTTAAATCAGTTTAAACGAATTCAAAGCTTGTTTATGCAAGTGTTTTGACAATGA
ATGTTCTCAAATCGATTGAAACTTTTTGGAGTTTATCACATCCACAGAAGAGTTTGAACATTTATTAGAAGCGCCAA
GGAGAGTTAAGACTGGTTTTTGGGCATTGATATCACGGTAGAGAAAATAAATTGGATTGGGATTTGATCATACAGGT
CTAACTACCGAACTGCTTTCTACCTACCAAAGACTTTTTGTTTAATTTGTAAAGCCCAAAACCTTGCAATCGAAGCC
CAAAACCTTGCAATCGAAGCCCAAGGGCTAGATAGATTAAATATCCGGTTGACAAAAGAAAAACGAACTAGCTTC
CAAAGAAAAAATCGTAAACATGTTAATCGGCAAAAAAAAAAAAAAAAAACAAGTGGCACGTGTCATTGTTGGGCA
AACGAGATGAGGTTGCCGTAACGTGTGGTCATCACACTTCTCTTCCTTCCTTCTTTCTTTCCACCACCTCCTTTCTT
CGTCCTCATCTCTTTCCTTCTCCGATCTATATTTAACTTCTTCGACGACTACGAAGATTAAAAATATCTAATCTTTTGT
GTTACCCACAAGAAAAGATATATATCTTTCTTTTATCTTCTTCTCTTGTACGATATTTTGCATGAGATTTTAAAACTCC
TCTTCTCACTCTCTGGTTTAGCTATG

BrSGR1 (XP_018512704.1) promoter
ATAGATAAGTTCCGACCGAAGCCGCAACGTACAGAATTGTTTCTAAATATTCGGCGAACCGCTTGTCTCTTTGTCCC
GAAAGGTAGTGTATTATCTGATAACGAATCGAAGAGAACTCGTCAACGCTACGTTTGTGCTTTAATGAGACGACAC
ATGCCAACTCATGCAGTGAAGAAGCTGGACACCAACGTGTAACTCACAGCTTCACCTCGTCAGAGCGGATTTTGG
ACTTGTGTAAAGTCTACAAATAGAAGCAACCTTCACTTTTCACTCGTATCGTAAGATTGTTGACTCCAACACGCAA
ACAACAAAAAGAAAAACAAAAAGAAAAACCTAATTTTCTGGAAGATAAAGCTTAACGGAAAGCGAAAGCAAGCT
CGTTTTTTTTAAGGGCTTCCAGCTTTTGATATTGTGGGTTTTACGAACGCGCAATAATAGCGTGAGACGTTAATATAG
TTTTTCTTTTATTTAAGGATTGTATTTTCCTTCAGTCTTGTTTTTACAGATACTGATATTGAAACGAAGCAGAGATG

BrDIN1 (XP_009150416.1) promoter
GCAGACACTAGGCAATAAAGGCACGATGACCCCGAGAGCTTGGTGCTAAGGATATACAGGAAGTACGCACTAGCA
GATGCCATTGTAGTTGTGGCCGCAAATAACGCAAAACGCCCGTTGGTCTTACTGACTCCAAAGGGTAAATTCCCTT
CCCCAAGCTGTGTACTCAAAGCTGTTACTAAGCCATACATAACAAACCCAATCACTGGCAAAGGAACACCTGGAG
AAATGCAAAAGAACAGTCACTTAGTTCAGCTTTCAACATGGAGCATGCTATTCATTGACACAGCTCATAGCACAGA
TTCAAGATTATATCGCATTGAGAGTGATTCAATCAGAATCTAATCATGCCTATGAGATCAAATACCATGAATACAAAT
CTTGAACTAAGCTACATACAAGTTTCCAGGTTAATCTAAGCTCAAGACTACATTAATCACCACTAGTGTATAGTACC
AAAAACAAGGGCGTAATCGCTGTTCAATACATCGCCGCAAGTACCACCACCAACGGGGCAAAAGGCATCGGAGCC
AGTGAGTTTCAGGTAGGTTAAATACGCGGTGTCTAACATCCCGATACCGCCGAGTCCGGTGAACCAGCTGTATGTC
GAACTGTTAATAGATACCGAATCTACACCATCATCCGACTCAGATGACGAGCATTTGATCGAAAATCTCCGGCGAG
AGACCTGATTAAGAACATCTGACTTCAATTAAACCAATCACACTACCATCACACACGAAAATGAAACGAGATTTAC
CTCGAAACGTCTTGGATTGAGAGACGACGACGAAGGAGGAAGCTCACGGAAGCCGAAACGAAACTGACAAGAC
GATACAGGGATAAACCTCGCCATCATCGTCGTCGTTGAACAATCGCTAATCCAACATCATCGAAAAGCTTCCCTCA
ACTTTCACTCCCTTCCGTTTCGTTAATGGGCCTAAGGCAAGGCCCATCTAGTATTAAGAGCTGGGCCTAATAAGAAA

CGTATAGTAGCATCTAATCCGAGTCAGCAAGTTATCCATATTACGACTTACGAGGTAACCGAGTTGGACTTATCAAC
TGATATCCATACTCCGCATGGCTTACATGTTGGACTCGGATAGATCTTCTAGAACTGGCTGACGTGTCAGTAGTTCC
ATATCCTTTGGCTTTGTGTCTGTATATATATACATAACATGCACGAGAAGTATAACTCAAAGTATCTACAAACACAAG
AATAAAAAATG
